# Medication self-management support for people with diabetes and low health literacy: A needs assessment

**DOI:** 10.1371/journal.pone.0232022

**Published:** 2020-04-24

**Authors:** Boudewijn B. Visscher, Bas Steunenberg, Eibert R. Heerdink, Jany Rademakers

**Affiliations:** 1 Healthy and Sustainable Living, University of Applied Sciences Utrecht, Utrecht, The Netherlands; 2 Division Pharmacoepidemiology and Clinical Pharmacology, Utrecht Institute of Pharmaceutical Sciences, Utrecht University, Utrecht, The Netherlands; 3 Research Department, NIVEL (Netherlands institute for health services research), Utrecht, The Netherlands; 4 Department of Family Medicine, Maastricht University, Maastricht, The Netherlands; Uppsala University, SWEDEN

## Abstract

**Introduction:**

An adequate level of health literacy is regarded as a prerequisite for adequate medication self-management. Low health literacy skills are relatively more common in people with Diabetes Mellitus type 2. The aim of this study was to explore the needs regarding medication self-management of people with type 2 diabetes and low (functional, communicative and critical) health literacy, and their preferences for medication self-management support.

**Materials and methods:**

A two-stage qualitative needs assessment study was performed using in-depth individual interviews and focus groups.

**Results:**

The participants preferred to be supported with reliable and easily understandable information, adequate interactive communication with health care professionals and fellow people with diabetes and tools for medication self-management support.

**Discussion:**

Future interventions should be created in co-creation with people with low health literacy and fulfill the expressed needs on information, communication and tools to improve self-management.

## Introduction

Diabetes Mellitus type 2 (DM2) is a complex and demanding chronic disease that requires extensive self-management [1]. Inadequate self-management can accelerate the onset of complications caused by DM2 and deteriorate the quality of life of people with DM2 [1,2]. The self-management activities in DM2 mainly focus on lifestyle and medication treatment [3]. Regarding medication treatment, self-management activities consist of e.g. measuring glucose, adjusting insulin dosage, adherence to oral antidiabetics (OAD) and dealing with side effects. This so-called ‘medication self-management’ is defined as the range of tasks people have to undertake to successfully manage their therapeutic regimen and sustain safe medication use [4]. Medication self-management requires a high level of control from a person and some autonomy to adjust his or her medication if necessary [5].

Health literacy is the ability of individuals to gain access to, understand and use information in ways that promote and maintain good health [6]. Health literacy consists of different sets of skills, as is described in the model of health literacy by Nutbeam (Table 1) [7]. People with low health literacy more often experience problems with misunderstanding on prescription medication labels and medication nonadherence [7,8,9]. Moreover, low health literacy skills are relatively more common in people with DM2 [10,11,12].

**Table 1:** Three types of health literacy.

Functional health literacy	“basic skills in reading and writing that are necessary to function effectively in everyday situations.” [7]
Communicative or interactive health literacy	“advanced cognitive and literacy skills which, together with social skills, can be used to actively participate in everyday situations, extracting information and deriving meaning from different forms of communication, and applying this to changing circumstances.” [7]
Critical health literacy	“advanced cognitive skills which, together with social skills, can be applied to critically analyze information and use this to exert greater control over life events and situations.” [7]

Interventions aimed at improving medication self-management are available [13,14]. These interventions have been proven effective, however seem to be too difficult to use and understand for people with low health literacy (e.g. the language in the presented information is too complex) [15,16]. A review focusing on multiple illnesses/chronic diseases highlighted the urgency for interventions tailored to the needs of people with low health literacy [15]. That’s why Rademakers et al. recommended involving people in all stages of intervention development (co-creation) [17].

A first step in co-creating an intervention is a needs assessment [18]. Previous studies have focused on the needs of people with DM2, but most do not measure the level of health literacy, except for the study of Fransen et al. that concluded that people preferred personal support rather than written information and there was heterogeneity in attitudes towards self-management [19]. However, that study focused on functional literacy only and indicated that the association between functional health literacy and self-management was not straightforward. The authors suggested assessing interactive and critical health literacy skills as well, since they may be better predictors for self-management [19]. In addition, the study by Fransen focused on self-management in general and gave little specific attention to adequate medication self-management, while medication is an important therapy option in the treatment of DM2 and people with health literacy often experience problems with medication self-management [7,8,9,19].

Therefore the aim of our study was to explore the needs of people with low (functional, communicative and critical) health literacy and DM2 regarding medication self-management and to explore the preferences for medication self-management support.

## Materials and methods

### Design

A two-phase qualitative study was performed involving in-depth individual interviews and focus groups. First, in-depth individual interviews were performed with people with DM2 and a low level of health literacy. Second, results from the interviews were further discussed in focus groups and preferences for diabetes medication self-management support were explored. The Institutional Research Board of the Department of Pharmaceutical Sciences of Utrecht University approved the study protocol. The study conformed to the provisions of the Declaration of Helsinki [20].

### Study setting and participants

#### Convenience sample

People with DM2 and low health literacy were recruited by means of a convenience sample from two pharmacies in Amersfoort in the Netherlands serving a total population of 28,000 people. There were two inclusion criteria: having DM2 and low health literacy. The first step was screening potentially eligible participants. In the Netherlands, people are registered at the pharmacy. The participating pharmacists extracted a list of people from the pharmacy information system that were dispensed the most common diabetes medication (metformin or insulin) at least once during the past year. The pharmacists selected people with low health literacy on the basis of potential risk groups (e.g. lower education level, higher age), statements known to be used to cover-up the lower level of health literacy (e.g. I have forgotten my glasses so I cannot sign the papers) and behavioral signals of not understanding information (e.g. no response to explanation of medicines). The potentially eligible participants were contacted by telephone by the pharmacists (starting at the top of the list) or contacted when visiting the pharmacy. The pharmacist informed them about the study in suitable, understandable language and asked permission to make an appointment for an intake interview with the researcher.

#### Health literacy level

In the intake interview the level of health literacy was determined by means of the Functional, Communicative and Critical Health Literacy scale [21]. The Functional, Communicative and Critical Health Literacy scale measures three aspects of health literacy, using 14 questions: functional (5 questions), interactive/communicative (5 questions) and critical (4 questions). All questions were scored on a four point Likert-scale (1–4) with a range from never perceiving difficulties too often perceiving difficulties. Mean total and mean sub-scale scores of the Functional, Communicative and Critical Health Literacy scale were calculated by summing items scores and then dividing the sum score by the total number of items (in total or in sub-scale). Based on previous research, potentially eligible participants with a mean score ≤3 in total or on a sub-scale were defined as having limited health literacy and were included in the study [21,22].

#### Informed consent

The informed consent was written in an easy and understandable language, and additional information was given and questions were answered during the intake interview. All included participants signed a written informed consent form. To create a relationship of trust between the researcher and the participants, two meetings were planned before the interview and/or focus groups. The first encounter was the intake interview at the pharmacist and the second one was a phone call a week before the interview and/or focus group, to see if there were any ambiguities about the planned interview and/or focus group and to hear if the participant would like to discuss specific topics. Trust between the researcher and the participants was necessary so that the participants could freely communicate about perceived barriers and needs [23].

### Interviews and focus groups

#### Individual interviews

**Fig 1:**
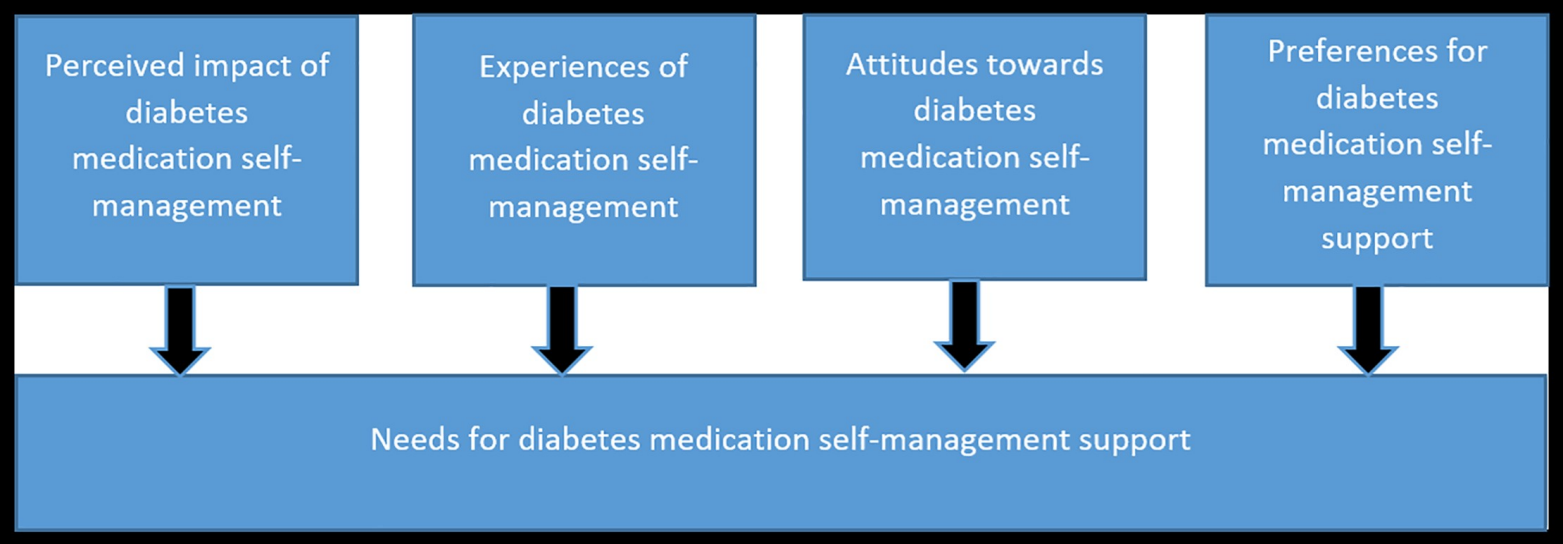
Framework for ordering person’s needs for diabetes medication self-management support.

The interviews were conducted at the participant’s home to develop the relationship of trust in an informal setting. A topic list was developed to explore the perceptions, barriers and needs. The topic list (S1 Table. Topic list interview) was based on a framework used the study of Fransen et al. that measured the needs of people with DM2 [19], which was guided by the results of a literature review [24]. To adapt the framework to our study, self-management was changed to medication self-management. The framework included the following categories: perceived impact of diabetes medication self-management, experiences of diabetes medication self-management, attitudes towards diabetes medication self-management and preferences for diabetes medication self-management support (Fig 1). The interview process was iterative and was performed by one of the researchers (BBV). The recruitment of participants by the pharmacists, the intake interviews and the interviews was done simultaneously. The total number of interviews was based on data saturation, meaning that when a new interview did not lead to more information related to the research question, the recruitment of participants for the interviews was stopped. Two researchers independently (BBV and BS) determined whether the data saturation had been reached by discussing if the interview has led to more information related to the research question.

### Focus group

The focus groups were used to explore the perceptions, barriers and needs. Two focus group meetings were organized: one focus group with a part of the participants who also did the interview by drawing random numbers that corresponded with participants by an independent researcher (BS) and a second focus group with participants that did not participated in the first phase to gain new insights. Recruitment of these participants was conducted in the same way as recruitment of the participants that participated in the interview and they were recruited from the same population. The focus groups were conducted in a room at the University of Applied Sciences Utrecht (Amersfoort location). The focus group meetings consisted of three phases. First, the topics of the topic list (Annex A) were discussed. Second, the main outcomes of the interviews were discussed, to gain more insight into the previously given answers. Third, existing interventions to improve medication self-management were shown and discussed. These interventions were not tailored to people with low health literacy. The existing interventions were the book “I have diabetes, what can I do?” [25], MySuggr App [26] and Appsuline [27]. The existing interventions were intended as a starting point to broaden and deepen the conversation and to explore preferences in medication self-management support.

### Analysis

Both the interviews and focus groups were audio recorded and transcribed verbatim. The focus groups were also recorded on video. The Atlas.ti 8 software program was used for the management and analysis of the transcripts. The analysis proceeded through three stages, consisting of open, axial, and selective coding with constant comparisons taking place throughout each phase. In the selective coding phase, the codes were placed in the framework based on Fransen (Fig 1). The analysis was done by two researchers independently (BBV and BS), and where differences occurred, consensus was reached through discussion with a third researcher (JR).

## Results

### Characteristics of participants

For the interviews and focus groups, 21 potentially eligible participants were recruited by the pharmacist and they had an intake interview with the researcher. Three potentially eligible participants with a mean score >3 in total or on a sub-scale on Functional, Communicative and Critical Health Literacy scale did not meet the criteria of low health literacy and were excluded. In total 18 participants participated in the study: 7 participants participated in the interviews, 6 participants participated in the interviews and focus group and 5 participants were recruited for the focus group only.

Table 2 shows the background characteristics of the participants. Most participants were male (11/18) and the age of the participants varied from 40 to 79 years old. Most of the participants had a Dutch ethnic background (15/18) and those with a migration background live for more than 30 years in the Netherlands. On the functional health literacy scale, 14 participants had low health literacy skills and can be considered low literate.

**Table 2:** Background characteristics of participants with diabetes type 2 and low health literacy (n = 18).

** **					Functional, communicative and critical health literacy scale—mean scores^a^			
Gender	Age	Migrant Background	Years since DM2 diagnosis	Inject Insulin	Functional	Communicative	Critical	Mean
Male	64		10		2.8	2.8	2.5	2.7
Female	67		8	x	2.4	3.2	4.0	3.2
Female	64		10	x	2.2	2.4	2.0	2.2
Male	73	X	23	x	1.6	2.6	1.0	1.7
Male	67		35	x	1.4	1.6	1.8	1.6
Male	77		20		3.0	2.6	1.5	2.4
Female	79		18	x	2.8	2.8	1.7	2.4
Male	53		17	x	2.2	1.0	1.0	1.4
Female	74	X	26	x	2.8	2.8	3.0	2.9
Female	43		14		3.6	2.4	2.0	2.7
Male	40		2		1.0	1.0	1.0	1.0
Male	68		18	x	1.8	1.8	2.0	1.9
Male	69		9	x	2.0	2.8	1.0	1.9
Male	48	X	12		2.8	2.4	2.0	2.4
Male	66		14		3.2	2.8	2.8	2.9
Male	65		15		1.6	1.4	1.0	1.3
Female	79		16		2.4	2.0	1.8	2.1
Female	60		10	x	4.0	2.6	1.0	2.5

^a^ Range score 1–4. Mean score ≤3 in total or on a sub-scale were defined as having limited health literacy and were included in the study

### Perceived impact of diabetes medication self-management

The participants described that their lives had barely changed since the diabetes diagnosis. Especially on a day at home following a daily routine, the participants hardly perceived any impact of their illness and their medication intake. The participants experienced difficulties with medication self-management when they changed their daily routine, for example when leaving home for a visit. In such cases they had to think of many things to take with them (medicines, nutrition), which cost them a lot of energy. Participants expressed the need to make it easier to remember all the necessary things when changing their daily routine.

*‘‘Taking medication is not always easy*, *especially with that insulin*. *Especially when we go to someone in the evening*, *we forget it*. *In the morning we are always at home*, *but if we go to dinner with someone at night I forget the insulin*.*”*

***‘‘****Well when I eat at a table*, *I have my medication in sight and then I know that I have to take my medication”*

### Experiences of diabetes medication self-management

Initially, most participants stated that they strictly adhered to their medication schedule and that they did not need additional support. After additional in-depth questions about the way they adhere to their medication schedule, it became clear that they did experience problems with their adherence. The participants who used insulin explained that the amount of insulin they inject depended on their overall feeling, without measuring the actual blood glucose values. Reasons for injecting insulin without measuring blood glucose values varied: they did not want to measure, it hurt when inserting a needle or it did not make sense because ‘‘the blood glucose values are always the same”. Some expressed the desire for monitoring blood glucose values without having to prick.

All participants linked their medication intake (OAD, short and long acting insulin) to their daily eating and sleeping rhythm (e.g. before breakfast, during dinner and before going to bed). The participants had different ways of ensuring themselves that the medicines were taken. A tool to preserve, distribute and organize the medicines was a frequently expressed method. These systems could be supplied by the pharmacy in the form of prefilled packages, or through use of their own medication boxes. These medication boxes were bought at a pharmacy or created by the participants themselves. Especially the insulin was put in a visible place, so that they did not forget it. Some participants were helped by their partner or informal caregivers to remember the medication intake. Many participants used an alarm clock for taking medication.

*“I make boxes with pills for the entire week for my husband and also for me*”

*“On the basis of how I feel I can judge whether I am high or low in my sugar and therefore I do not measure glucose*”

*“I do not measure my blood glucose before injecting insulin*, *because my blood glucose values are often the same*”

### Attitudes towards diabetes medication self-management

In the selective coding phase, there was a wide range of attitudes towards diabetes medication self-management. All codes were printed and two researchers discussed the codes to formulate subgroups from the codes. The discussion resulted in that the codes in this category could be subdivided into three groups of attitudes towards diabetes medication self-management. Every participant fitted into one of these groups.

#### Adequate self-management

The first group consisted of participants reporting that they had adequate medication self-management and were motivated because they wanted to live as long as possible with as few complications as possible. They have also changed their lifestyle (nutrition and exercise) after the diagnosis.

#### Unaware

The second group included participants with an unaware attitude to medication self-management who wanted to keep charge of their own life, lifestyle and the amount of taken medication. Most of the participants in this group expressed that they did not understood the relationship between medicines, diabetes and their lifestyle.

#### Aware but not activated

The third group of participants were aware of the importance of adequate medication self-management but did not know where to start or how they could adequately self-manage their medication.

*“I am not very good at taking medication on time*. *I use metformin five hundred milligrams three times a day*, *but I sometimes forget* … *and I do not know how I can always take my medication* …*”*

*“I know it is good to always take my medication*, *I tried*, *but I just don’t succeed*.*”*

#### Participants’ preferences for diabetes medication self-management support

**Fig 2:**
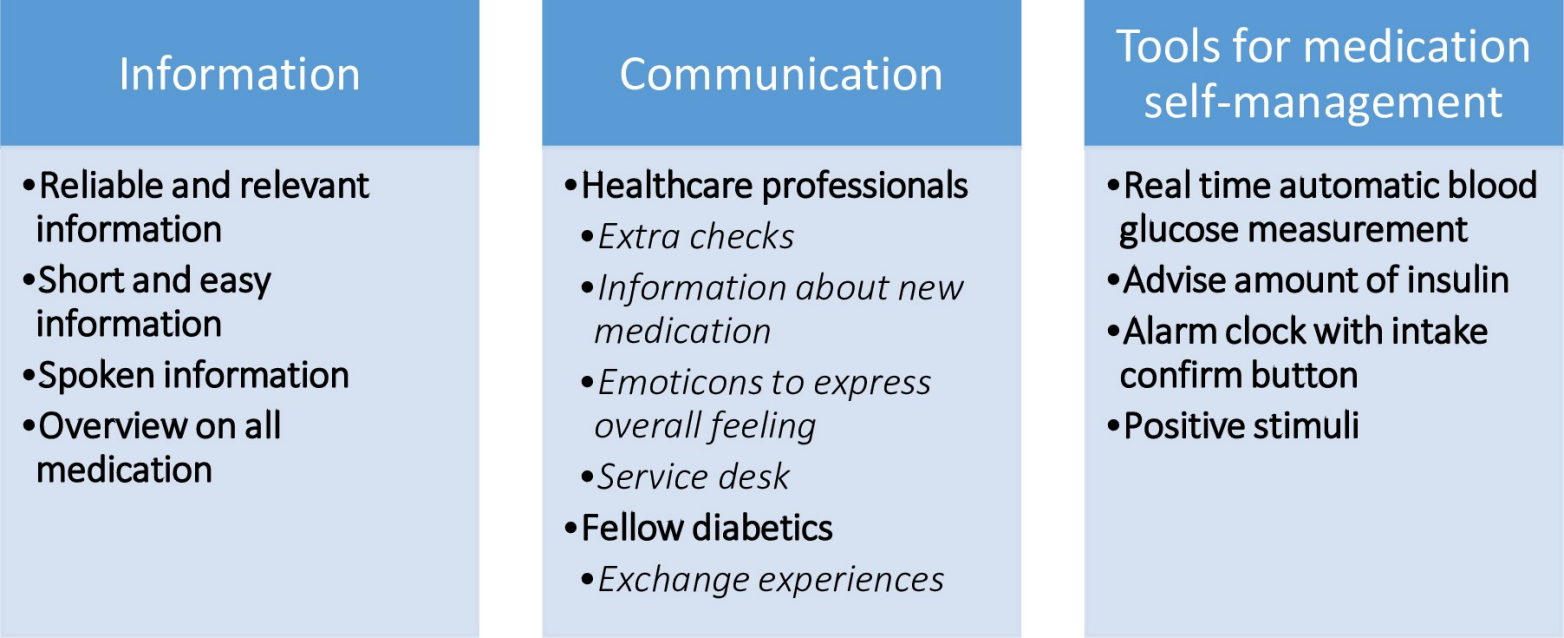
Participants’ preferences for diabetes medication self-management support.

Participants found it hard to distinguish the medication self-management preferences from other preferences in self-management support. For completeness these preferences are shown, partly because they have a relationship with medication self-management support. All codes were printed and two researchers discussed the codes to formulate subgroups from the codes. The preferences for support can be divided into 3 categories: preferences for information, communication and tools for medication self-management (Fig 2).

#### Information

The participants found it difficult to read and understand the information on the labels of the medication package and medication prescription due to the small print and difficult words. A part of the participants were satisfied with the current support of the healthcare professionals. According to the participants, the current support consisted mainly of presenting or giving information. The participants expressed that the medication information was clearly presented by their healthcare professional and that they could easily ask them questions if something was unclear. Besides the information from their healthcare professionals, participants received information about medication self-management from other sources: the internet (mainly through the first hits on google.com), the Dutch diabetes association, or family. They found it difficult to estimate whether the information from other sources than the healthcare professionals are relevant and reliable and therefore they trusted mainly the information of the healthcare professionals and tried to follow those instructions for medication self-management. The participants would like to have more information about medication, side effects and new available medicines. The information should be short and easy to read and some participants preferred spoken information / animations in multiple languages, because they experienced reading difficulties.

In the focus groups, there was a heterogeneity in preferences in presenting information: some participants preferred a booklet with information (e.g. ‘‘I have Diabetes, what can I do? [19]”), and others preferred an application. They preferred a simpler version of an application as was shown with less written information. Most participants had other diseases besides DM2 and they also had medication for those diseases. The participants expressed the feeling that they found it hard to distinguish which medication was used for exactly which disease. The participants preferred to have an overview of all the medication, including an overview of contraindications and side-effects of the medicines on other medicines.

#### Communication

The participants distinguished between communication with the healthcare professionals and fellow people with diabetes. The participants expressed the feeling that they preferred to be informed by the healthcare professional when new medicines were available and to have extra checks to see if the medication was taken properly. It was suggested in the focus group that including self-report on daily mood to an app. For example adding emoticons to the apps would be of added value, which can be discussed during the check-ups with healthcare professionals. Some participants would like to be advised by a 24-hour general service desk, which addresses all their medication related questions. Others did not want this because they preferred to have one healthcare professional that they trust and who already knows their background and medical history. The participants discovered in the focus group how valuable it is to be in contact with fellow people with diabetes. They would like to meet fellow people with diabetes more often to exchange experiences and tips about how to live with diabetes.

#### Tools for medication self-management

Some participants found information on a recently developed real time, automatic blood glucose measurement system and would like to have such system as a tool for their medication self-management. The system has recently become available but is often not reimbursed by the health insurer and none of the participants had tried the system. The blood glucose values can be displayed online, whereby they preferred to receive advice about the amount of insulin they need to take. Some participants preferred for the alarm clock to be set remotely by the healthcare professional. An experienced difficulty of the alarm was that users snooze or turned off the alarm, because of being busy with other things (e.g. having a conversation), and then did not remember whether they had the medication. The participants indicated that it could be useful to confirm to the alarm that the medication was taken. This would also create an overview of the taken medication. There was an ambiguous preference for obtaining positive stimuli: some would like it if good glucose values or walking enough steps were rewarded with positive stimuli (e.g. short positive messages), others found it rather irritating.

*“Look*, *if they have something new*, *I would like to know*. *If they have new medication with fewer side effects or something*, *then I am interested*. *Something new can also be good*, *I am interested in that*.*”*

*“When I get new medicines my daughter says*, *you really have to take breakfast*. *She really has to explain it in Arabic*. *I cannot read and understanding other languages than Arabic is difficult for me*.*”*

## Discussion

This study explored the needs and preferences of people with DM2 with low health literacy regarding medication self-management and the preferences for medication self-management support. The participants differed in their needs, attitudes and preferences. With respect to attitudes towards diabetes medication self-management, three groups could be discerned: adequate self-management, unaware and aware but not activated. The preferences for support could be divided into three categories: preferences for information, communication and tools for medication self-management.

This study highlighted additional needs and preferences of people with DM2 and low health literacy, which will be used in the development of an co-created intervention in the next phase of this project. In the development of the intervention, options to personally modify and tailor the intervention may be important to create an optimal fit between the intervention and the needs and preferences of the user of the intervention [15,17]. For example, the three distinguished groups on attitude could function as a persona to tailor the preferences and needs for improving medication self-management. In addition, consideration should be given to mechanisms and factors that influence medication self-management. The self-determination theory emphasizes the importance of the underlying reasons for behavior. The self-determination theory indicates that skills and knowledge are not sufficient to change behavior, but that autonomous motivation is needed [28]. This autonomous motivation can increase in various ways, whereby the preferences differ per person [29]. When developing an intervention, the various routes to increase autonomous motivation must also be studied and taken into account.

The added value of patient engagement in the development of interventions is increasingly recognized and valued, but there is little literature on how to best involve people with low health literacy [30,31]. A strength of our study is the recruitment and involvement of people with low health literacy in this study, and there are a number of possible success factors for involving people with low health literacy.

First, the people with low health literacy and the self-management problems should be noticed by the healthcare provider. At the start of the interviews, most of the participants expressed that they have an adequate level of medication self-management. However, additional questions (on e.g. medication adherence, understanding prescription labels) showed that the level of medication self-management was often insufficient. This discrepancy could contribute to the fact that healthcare professionals not sufficiently notice and recognize problems with self-management and that care users perceive no need to ask for help since they regard their medication self-management as adequate. Health professionals should be better trained in identifying people with DM2 with low health literacy and problems with medication self-management. There are tools available for this, for example the RALPH interview guide (Recognizing and Addressing Limited Pharmaceutical literacy) [32]. In this study motivated and experienced pharmacists participated, who were personally involved and selected the respondents. This surely increased the number of included people.

A second possible success factor, was the importance paid to the relationship between the researcher and participants, so the participants feel free and secure to communicate about perceived barriers and needs [23]. The frequent contact prior to the interview (intake interview and telephone conversation a week before the interview / focus group) and the interviews that were conducted at the participant’s home, created an informal setting in which participants could communicate their perceived barriers and needs for medication self-management. The researcher was especially trained to communicate in line with the level of this subgroup. This relationship of trust was initiated by their own pharmacist when introducing the researcher to the respondents. In the interviews, the people also indicated that they trusted their pharmacist, which increased the likelihood that they will also trust the introduced researcher [33].

A third possible success factor for involving people, were the focus group meetings. Initially, the participants found it very difficult to express needs and preferences for support. The focus group meetings were helpful in expressing needs and preferences for improving medication self-management with the suggestions of other participants and the shared interventions. In addition, the participants also preferred to have more contact with fellow people with diabetes in general. In other studies, such contact with fellow people with diabetes empowered people and improved their self-management [34,35]. In Dutch healthcare there are already opportunities to have more contact with fellow people with diabetes. In the focus group setting we did discuss the added value of peer group support, no one expressed that they ever attended structured group education. It could be that the participant does not know that there are peer groups available or that they are not yet found by the target group. Awareness of the existence of such peer groups should be increased and healthcare professionals could better inform persons with low health literacy, or even better introduce them to a peer group.

The pharmacists selected participants on potential risk group, verbal statements and behavioral signals of not understanding. A pre-condition for this kind of selection is that the participants visits the pharmacy and are known to the pharmacist, which can make the results less representative for the target group at a whole of people with low health literacy skills (who might not come to the pharmacy). However, this convenience sampling method was a successful strategy of involving this hard-to-reach target group in research. Another limitation in this study is that the persons with low health literacy find it difficult to think in concepts and express their feelings in concrete themes and needs. To increase the reliability of the results, a relationship of trust between the researcher and participants was created. However, there is still a possible bias in the interviews and focus group meetings that the participants have given socially desired answers about their barriers, needs and preferences. The focus group meetings were helpful in expressing needs and preferences with the suggestions of other participants and the shared interventions. Further research to gain more insight in how to explore the needs of persons with low health literacy and how to search for an adequate way to co-create with persons with low health literacy is needed. Another limitation of this study is that we only included people with DM2. We have deliberately opted for people with DM2 because they generally got DM2 later in life and had to adopt a different lifestyle and learn medication self-management skills. Medication self-management is equally important in people with Diabetes Mellitus type 1, and this study warrants repetition in that population.

The results of this needs assessment will be used to develop a medication self-management intervention that addresses the great heterogeneity in needs and preferences and will be developed in co-creation with people with low health literacy and DM2 using the intervention mapping method [18].

## Supporting information

S1 Table Topic list interview. (PDF)
