# Supplementary material for: Medication self-management support for people with diabetes and low health literacy: A needs assessment
Source: PLoS One. 2020 Apr 24;15(4):e0232022. doi: 10.1371/journal.pone.0232022 (PMC7182204; doi:10.1371/journal.pone.0232022)
Supplement: S1 Table — (PDF) [file pone.0232022.s001.pdf]

S1 Table. Topic list interview

Perceptions	o Expercienced effect medicine use<br>o Frequenty medicine use<br>o Type medicine<br>o Management of medication<br>o Obtained information medicine use<br>o Help with medication inake
Barriers	o User-friendly intake of medicines<br>o Dosing schedule<br>o Forgotten medication intake<br>o Understanding usefulness and necessity of medication<br>o Fear of side effects
Needs	o Internet /apps<br>o Tools<br>o User-friendly information<br>o Information
